# Patient-Specific Method of Generating Parametric Maps of Patlak *K*_*i*_ without Blood Sampling or Metabolite Correction: A Feasibility Study

**DOI:** 10.1155/2011/185083

**Published:** 2011-09-07

**Authors:** George A. Sayre, Benjamin L. Franc, Youngho Seo

**Affiliations:** ^1^UCSF Department of Radiology and Biomedical Imaging, University of California, San Francisco, CA 94143-0628, USA; ^2^Division of Nuclear Medicine, Radiological Associates of Sacramento, Sacramento, CA 95815, USA; ^3^UCSF Department of Radiation Oncology, University of California, San Francisco, CA 94115-1708, USA; ^4^UCSF Helen Diller Family Comprehensive Cancer Center, San Francisco, CA 94143-0875, USA; ^5^UC Berkeley & UCSF Graduate Program in Bioengineering, Berkeley and San Francisco, CA 94158-2330, USA

## Abstract

Currently, kinetic analyses using dynamic positron emission tomography (PET) experience very limited use despite their potential for improving quantitative accuracy in several clinical and research applications. For targeted volume applications, such as radiation treatment planning, treatment monitoring, and cerebral metabolic studies, the key to implementation of these methods is the determination of an arterial input function, which can include time-consuming analysis of blood samples for metabolite correction. Targeted kinetic applications would become practical for the clinic if blood sampling and metabolite correction could be avoided. To this end, we developed a novel method (Patlak-P) of generating parametric maps that is identical to Patlak *K*_*i*_ (within a global scalar multiple) but does not require the determination of the arterial input function or metabolite correction. In this initial study, we show that Patlak-P (a) mimics Patlak *K*_*i*_ images in terms of visual assessment and target-to-background (TB) ratios of regions of elevated uptake, (b) has higher visual contrast and (generally) better image quality than SUV, and (c) may have an important role in improving radiotherapy planning, therapy monitoring, and neurometabolism studies.

## 1. Introduction

PET with ^18^F-fluorodexoyglucose (FDG) has become a mainstay in the detection, initial staging, restaging, prognostication, treatment monitoring, and treatment planning for a variety of cancer types [1, 2]. FDG-PET is effective in studying a wide range of cancers, because most tumors are hypermetabolic due to the Warburg effect, a biochemical process where cancer cells preferentially use glucose metabolism rather than oxidative phosphorylation. The purpose of upregulating glucose metabolism is to support a microenvironment that is toxic to normal cells but allows tumor cells to elude apoptosis [3, 4] and initiate local invasion and metabolic spread [5]. Two proteins are mainly responsible for this increase in glucose metabolism: glucose transporter (GLUT) protein to transport glucose across the tumor's membrane and hexokinase to phosphorylate it in preparation for glycolysis [6]. Another important protein, glucose-6-phosphatase, is responsible for dephosphorylating glucose but has low activity in tumor cells. Because FDG is an analog of glucose, it enters tumor cells via GLUTs and is phosphorylated by hexokinases. However, it accumulates as FDG-6-phosphate because its slightly different biochemistry prevents it from being metabolized further down the glycolytic pathway. 

Dynamic FDG-PET provides a means of quantifying GLUT and hexokinase expression. Using the two compartmental model for FDG, GLUT expression is quantified with *K*_1_, and hexokinase expression is quantified with *K*_3_ [7]. However, compartmental models require nonlinear regression, and evaluation at the voxel level is difficult [8]. Late-time graphical analysis techniques of FDG-PET such as Patlak and simplified kinetic analysis (SKA) methods are more robust to noise and may be used to generate parametric maps of the net influx rate constant, *K*_*i*_ [9]. The main difference between the two methods is how the arterial input function (AIF) is determined SKAs utilize population-based average AIFs whereas AIFs are acquired on a per-patient basis for Patlak. In addition, SKAs require fewer intrusive blood samples than Patlak and perform almost as well in practice given strict adherence to injection and acquisition protocols [9, 10]. However, Patlak may be the more robust method if the population-based AIF does not account for variation in large patient populations. Furthermore, population-based AIFs have only been developed for FDG, so at present, Patlak is the only option for graphical analysis of other tracers with irreversible uptake, such as methionine (MET) and fluoro-L-thymidine (FLT). 

In contrast to dynamic analyses, the standardized uptake value (SUV), which is the widely accepted standard for PET, only requires a static scan, is easily implemented in the clinic, does not require determination of an AIF; and may be used to perform whole-body screens to detect tumors and metastases [11]. SUV approximates Patlak *K*_*i*_ by assuming that (a) the unmetabolized component of tracer is negligible at late times, and (b) the ratio of injected dose to body weight is proportional to the area under the curve (AUC) of the AIF [12]. These conditions are not always met, and when they fail, SUV values can suffer [12]. In fact, several studies have shown that the inclusion of compartmental modeling and/or Patlak *K*_*i*_ leads to improved treatment monitoring and prediction [13, 14] and tumor staging and differentiation between tumor and benign lesions [15–18]. In addition, it has been shown that biological tumor volumes (BTVs) derived from FDG-SUV maps are larger than those derived from *K*_*i*_ maps [19] due to contrast improvement of the tumor from the background on the *K*_*i*_ map [20]. Thus, Patlak *K*_*i*_ analysis may have a significant impact on radiotherapy planning by (a) limiting the amount of normal tissue exposed to high radiation dose and (b) improving dose maps to better reflect the kinetic heterogeneity of the tumor [21]. 

In the clinic and in research studies, arterial blood sampling is an intrusive and potentially risky process that necessitates specific expertise to manage added complexities [22]. These findings are confirmed in our own experience at UCSF where maintaining arterial line insertions and correcting for metabolites are known problems. Thus, the goal of the present research was to develop a technique to generate parametric maps that are equivalent to or meet the same clinical objectives as Patlak *K*_*i*_ without arterial blood sampling or metabolite correction. The ultimate aims of this research are (a) to facilitate a wider use of dynamic PET studies using irreversible tracers such as FDG, FLT, MET, and acetate and (b) to improve radiotherapy planning by providing better representations of tumor boundaries through Patlak *K*_*i*_ parametric maps. In this manuscript, we describe the methodology of our technique (Patlak-*P*), and perform quantitative and qualitative comparisons of it with Patlak *K*_*i*_ and SUV for several FDG and FLT studies as a means of initial validation. In addition, we outline the possible impact of Patlak-*P* in radiotherapy and other application areas such as treatment monitoring and neurometabolism studies.

## 2. Methods

### 2.1. Patient Data

This work utilized patient data sets that had been collected as part of a larger human subjects imaging trial that was approved by our institutional review board (IRB). Each patient was suspected of having oropharyngeal carcinoma and underwent both FDG-PET and FLT-PET imaging studies as part of the larger trial.

### 2.2. Patient Preparation

Three patients, each of whom was imaged using FDG-PET and FLT-PET, were required to complete a consent form before their studies began. Following the insertion of the intravenous catheter, a customized radiotherapy mask was then prepared, and the patient was asked to void their bladder.

### 2.3. Cardiac Acquisition

Each patient was asked to lie flat on the carbon fiber bed of a Siemens Biograph 16 PET/CT scanner. A topogram was then acquired in the craniocaudal direction to assess the patient's overall radiographic profile. A computed tomography scan for attenuation correction (CTAC) of the heart was then obtained in the caudocranial direction with the following settings: 80 mA current, 0.75′′/rotation, 27 mm rotation, and 5 mm recon slice thickness. Subsequently, an 11-minute dynamic PET scan with a cardiac field of view (FOV) was begun simultaneously with tracer injection: 10 mCi FLT or 15 mCi FDG. The images were reconstructed using ordered subsets expectation maximization (OSEM) with 8 subsets and 4 iterations. One blood sample was drawn at the end of the cardiac PET acquisition. The purpose of this dynamic scan was to acquire the initial dynamic profile of the AIF.

### 2.4. Head and Neck Acquisition

Before imaging, each patient was fitted with their radiotherapy mask. A topogram was then acquired in the craniocaudal direction to assess the patient's overall radiographic profile. Following acquisition of the topogram, a CTAC of the head and neck was then obtained in the caudocranial direction with the following settings: 80 mA current, 0.5′′/rotation, 24 mm rotation, and 5 mm recon slice thickness. Subsequently, a 45-minute dynamic PET scan of the head and neck (HN) was acquired after matching the CT FOV and recon slice thickness. The images were reconstructed using ordered subsets expectation maximization (OSEM) with 8 subsets and 4 iterations. Four blood samples were drawn at 15, 25, 40, and 55 minutes after-injection. The purpose of this dynamic scan was to acquire the late-time dynamic profile of HN tumors and their surrounding structures for Patlak analysis.

### 2.5. Patlak-*P* Methodology

The theory of Patlak-*P* is closely aligned with Patlak's original method [23]. At late times *t* > t*, the tracer concentration in exchangeable compartments is directly proportional to the tracer concentration in the plasma for irreversible tracers such as FDG. The well-known Patlak equation (1) is obtained by applying this assumption to first-order tracer kinetics

(1) $$\frac{P\left(t\right)}{\text{AIF}\left(t\right)}=K_{i}\frac{\int_{0}^{t}\text{AIF}\left(t^{\prime }\right)dt^{\prime }}{\text{AIF}\left(t\right)}+V.$$

Equation (1) states that *P*(*t*), the concentration [Bq/mL] of an irreversible tracer at time *t*, is described by two kinetic components: *K*_*i*_ (the net influx rate) and *V* (the total distribution volume). The major issue in calculating *K*_*i*_ is determining the complete time course of the AIF, which requires either (a) approximately 1 hour of invasive blood samples or (b) some combination of blood samples and an initial cardiac acquisition as we've done in this study. Though our protocol reduces the total number of blood samples needed, it is still intrusive and requires metabolite correction for FLT and other tracers including methionine. 

We addressed this problem by making a simple, yet novel assumption regarding the late-time sequence of AIFs. Fitting AIFs with a triple exponential function is an established method in reducing the effect of noise on kinetic calculations [24]. However, to our knowledge, the well-mixed assumption, which is often used in drug pharmacokinetics studies, has not yet been applied to dynamic PET. The well-mixed assumption, as applied to the AIF, is mathematically equivalent to simple exponential decay. By applying this assumption, late-time PET kinetics may be described without knowledge of the input function  (2)

(2) $$\begin{array}{cc} & P\left(t\right)-P\left(t_{0}\right)\\ & =\left(K_{i}\overset{t}{\underset{t_{0}}{\int }}\text{AIF}\left(t_{0}\right)e^{-\lambda (t^{\prime }-t_{0})}dt\prime +V*\text{AIF}\left(t_{0}\right)\left(1-e^{-\lambda (t-t_{0})}\right)\right). \end{array}$$

Equation (2) describes late-time kinetics relative to an initial dynamic frame at *t*_0_, which differs from the usual Patlak view. By evaluating the integral and performing algebraic rearrangement, (2) is transformed into an equation that does not contain any discernable kinetic parameters (3)

(3) $$P\left(t\right)=P\left(t_{0}\right)+\alpha \left(1-e^{-\lambda (t-t_{0})}\right).$$

The parameter *α* in (3) reflects the uptake between *t*_0_ and infinity and is a function of AIF(*t*_0_), *K*_*i*_, *λ* (AIF decay constant), and *V*. Inspection of (3) reveals that *P*(*t* = *∞*), or *P*_*∞*_, is equivalent to *P*(*t*_0_) + *α* and is directly proportional to *K*_*i*_ (4), as Patlak previously demonstrated [25]

(4) $$P_{\infty }=K_{i}\overset{\infty }{\underset{0}{\int }}\text{AIF}\left(t\prime \right)dt\prime =K_{i}\left(i{\text{AIF}}_{0}+\frac{{\text{AIF}}_{0}}{\lambda }\right).$$

Equation (4) states that *P*_*∞*_ is identical to *K*_*i*_ (within a global scalar constant) if we assume that the integral of the AIF is constant in space at late times. The global scalar constant, the total integrated activity (*i*AIF_*∞*_), is the sum of two terms: the total integrated activity from 0 to *t*_0_ (*i*AIF_0_) and the total integrated activity from *t*_0_ to infinity, (AIF_0_/*λ*). Thus, we now have a framework for obtaining parametric images that reflect the behavior of Patlak *K*_*i*_ without needing to determine an AIF or correct for metabolites. In addition, we have a basis for determining *K*_*i*_ given a priori knowledge of the input function such as population-based AIFs for FDG (see Appendix).

However, accurate calculation of *P*_*∞*_ maps is most dependent upon accurate estimates of *λ*, a global parameter and the only nonlinear term in (3). In contrast, *P*(*t*_0_) and *α* are linear terms and are easily calculated using linear least squares given knowledge of *λ*. To this end, we propose a strategy where *λ* would be determined first from nonlinear regression of a large region of interest (ROI) using the Levenberg-Marquardt algorithm [26]. By doing so, we only perform nonlinear regression on a time activity curve (TAC) with excellent statistics and subsequently linearize the model (3) for simple voxel-by-voxel fits. 

Since the biodistribution of tracers at late times changes minimally, the nonlinear noise properties of OSEM were assumed to have a minimal effect on predicting a simple, accurate weighting scheme for nonlinear and linear regression. Thus, we employed weighted nonlinear and linear regression to determine *λ* and *P*_*∞*_, respectively, with weights equal to the product of frame duration (Δ*t*) and the radioactive decay factor (DF). 

Because *P*_*∞*_ is just a measure of activity at very late times, it is easily set in the framework of SUV. Thus, we propose a new quantity, SUV_*∞*_, which better reflects the kinetic behavior of irreversible tracers such as FDG. The application of SUV_*∞*_ to treatment monitoring will be explored in the discussion section.

## 3. Results

### 3.1. Outline of Validation Steps

In our view, validating Patlak-*P* as a surrogate for Patlak *K*_*i*_ in radiotherapy planning required two major comparisons of tumors and other regions of elevated uptake derived from Patlak *K*_*i*_, SUV_*∞*_, and SUV maps, target-to-background (TB) ratios and qualitative assessments of image quality. TB ratios were calculated from manual segmentations of the target region and a nearby background region that presented the largest challenge to target visualization. In this way, TB ratios quantitatively reflected the ability of each technique to delineate the target from *all* nearby structures. The metrics for qualitative assessment were visual contrast, delineation of target boundary, and presence/absence of well-known structural detail such as tooth “holes” and the cerebellum. 

In an effort to maintain analytical cohesion, we decided to combine select quantitative and qualitative findings for each patient and present them in their own subsections. A final subsection was used to summarize general trends. 

 Tumor volumes derived from the three methods were not directly compared, because (a) there is no general consensus for which segmentation algorithm to use [27]; (b) dose maps drawn by radiation oncologists or nuclear medicine physicians are considered the gold standard [28]. Thus, the validation steps of the present work focused on image quality and contrast as it is reasoned that these factors primarily influence which regions are included in tumor volumes by therapy-planning physicians.

### 3.2. TB Ratios Methodology

**Figure 1:**
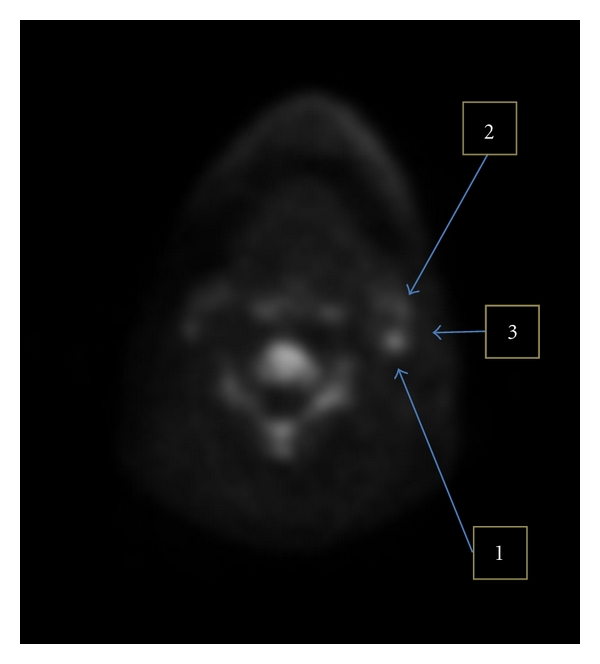
Representative example of target (1) and background definition (2) on SUV image from FLT-PET. The typical background (3) is less indicative of the difficulty associated with delineating the target.

As stated previously, the purpose of TB ratios was to compare how each technique is able to delineate tumors or other regions of elevated uptake from the background and adjacent structures. A representative example of how target and background definitions were defined is illustrated in Figure 1. 

### 3.3. Patient 1 Comparisons

**Figure 2:**
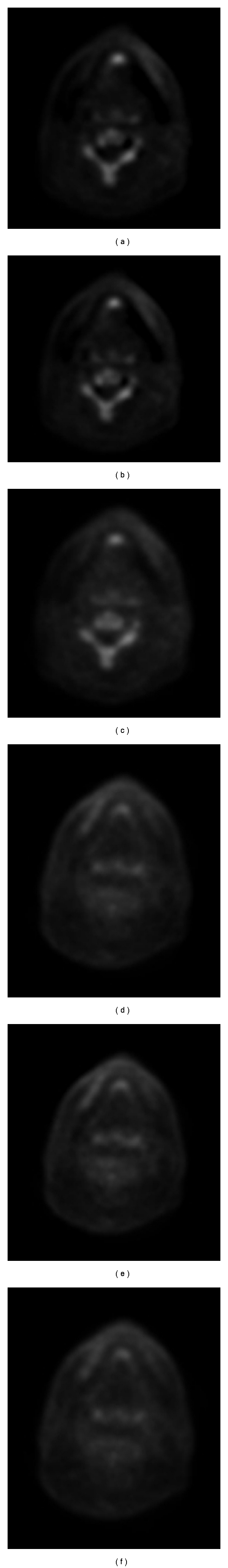
REU near base of the mouth as calculated by Patlak (left), Patlak-*P* (center), and SUV (right). Top row is FLT-PET, and the bottom row is FDG-PET.

Both FDG and FLT acquisitions of Patient 1 show a region with elevated uptake (REU) near the base of the mouth (Figure 2). 

Inspection of Figure 2 shows that FLT images derived from Patlak-*P* and Patlak have higher contrast than SUV and in general appear to be of higher quality because their backgrounds are diminished. These findings are quantitatively confirmed through TB ratios: Patlak-*P* (3.45), Patlak (3.77), and SUV (2.98). In addition, the REU appears to be slightly smaller in Patlak and Patlak-*P* images. Images generated from the FDG-PET scans are similar to their FLT counterparts, but the contrast differences are slightly less pronounced: Patlak-*P* (2.736), Patlak (2.481), and SUV (2.270). In this case, however, the Patlak-*P* parametric map has slightly higher contrast than Patlak. 

**Figure 3:**
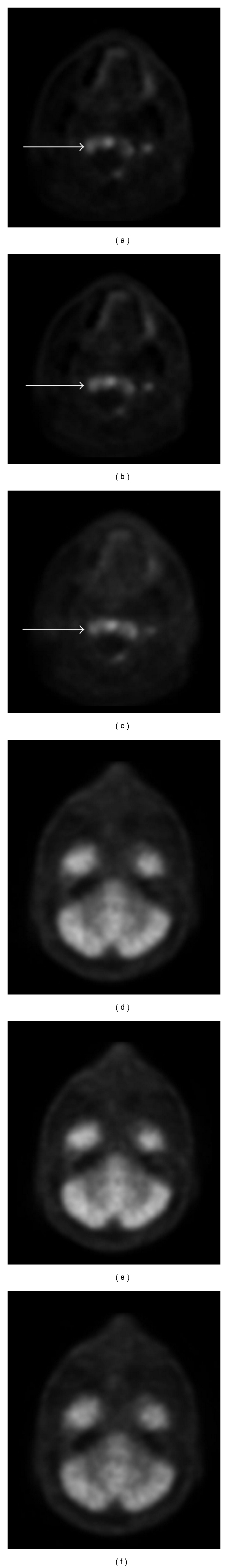
Transaxial slices of FLT-PET (a–c) and FDG-PET (d–f) that show difference in quality of visualizing well-known structures (teeth, cerebellum) for each technique: Patlak (left), Patlak-*P* (center), and SUV (right).

An overall analysis of image quality showed that parametric maps generated by the three methods follow the prior examples. For both FDG and FLT studies of this patient, Patlak and Patlak-*P* generated parametric maps with higher contrast and visual quality than SUV. Furthermore, this increased contrast led to sharper boundaries between regions of high uptake and their surrounding areas in Patlak and Patlak-*P* images, as evidenced in how well each technique resolved the teeth and bony structures (arrows) in FLT-PET (Figures 3(a)–3(c)) and the cerebellum in FDG-PET (Figures 3(d)–3(f)). In particular, Patlak-*P* predicts more uniform uptake in the cerebellum, when compared with Patlak and SUV. Such a result may have implications for neurometabolism studies (see Discussion Section). 

### 3.4. Patient 2 Comparisons

**Figure 4:**
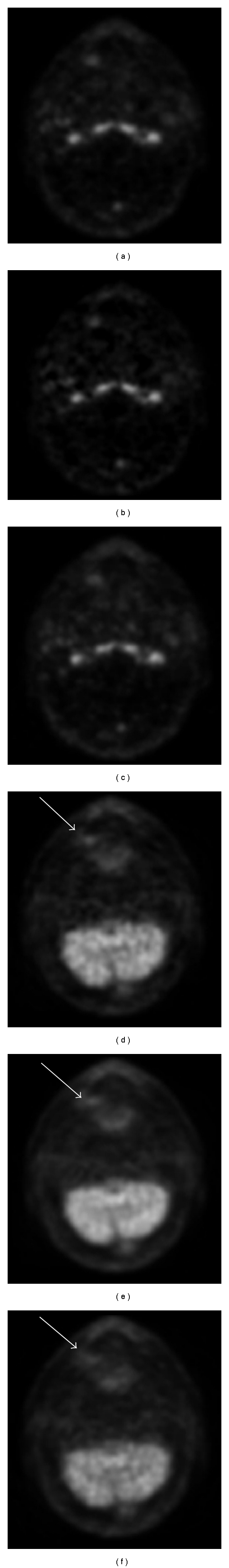
Region of high uptake near top row of teeth as calculated by Patlak (left), Patlak-*P* (center), and SUV (right). Top row is FLT-PET, and the bottom row is FDG-PET. Arrows indicate background ROI.

An REU was seen in both FDG and FLT images of Patient 2 near the upper row of teeth (Figure 4).

Inspection of Figure 4 shows that Patlak-*P* FLT images have higher contrast than either Patlak or SUV, which is mirrored by TB ratios of the REU: Patlak-*P* (5.64), Patlak (4.37), and SUV (3.71). Patlak-*P*'s high TB ratio is explained by increased diffusivity of the REU in Patlak and SUV images and increased background reduction in the Patlak-*P* image. In this study, however, the increase of image contrast is paired with a slight decrease in image quality.

The same behavior is observed in the FDG images, except in this case, the Patlak image has higher contrast and lesser quality than the Patlak-*P* image, though both are superior to SUV in both respects. The TB ratios do not mirror the relative performance of SUV: 1.92 (Patlak-*P*), 2.661 (Patlak), and 1.95 (SUV). It is more than likely that the TB ratio for SUV is improperly elevated, because the background ROI (indicated by arrows in Figure 4) appears to be improperly diffuse in the SUV image. In fact, it is much more difficult to visually separate the REU from the background region in the SUV image. Furthermore, from an overall visual perspective, the Patlak-*P* image appears to be higher quality than the Patlak and SUV images. This perspective is particularly in the soft tissue surrounding the REU and the cerebellum.

### 3.5. Patient 3 Comparisons

**Figure 5:**
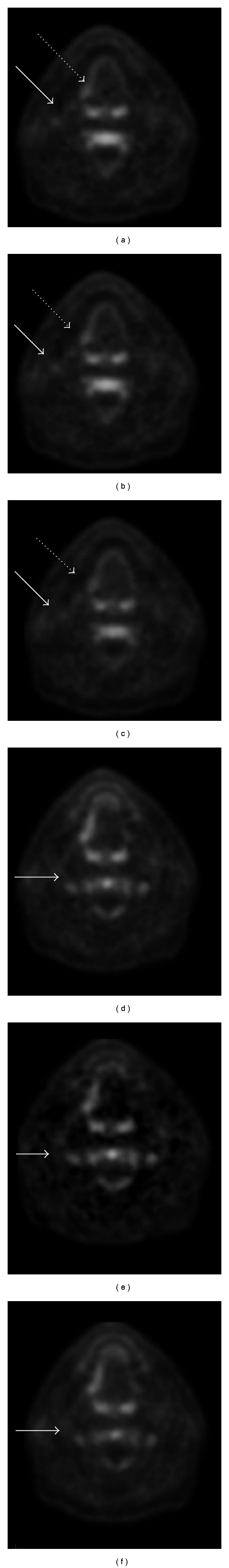
Caudal-most two slices of tumor as calculated by Patlak (left), Patlak-*P* (center), and SUV (right) from FLT-PET study of Patient 3. In the top row, dashed arrows indicate the tumor nodule, and solid arrows indicate the small REU. In the bottom row, arrows indicate a set of bony structures.

**Figure 6:**
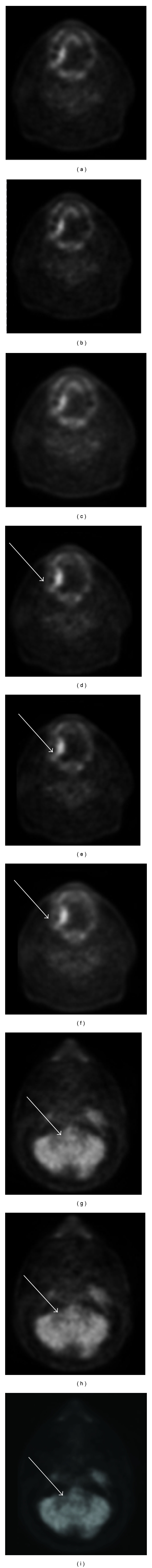
Caudal-most two slices of tumor (a–f) and cerebellar slice (g–i) as calculated by Patlak (left), Patlak-*P* (center), and SUV (right) from FDG-PET of Patient 3. Solid arrows indicate background ROI (d–f) and cerebellar region of interest (g–i).

A tumor was found on the tongue of Patient 3 and could be visualized in Patient 3's FLT and FDG images.  Figure 5 illustrates the caudal-most two slices in which the tumor is present in the FLT image, and Figure 6 illustrates the caudal-most two slices in which the tumor is present in the FDG image. 

The results shown in Figure 5 are similar to those seen in the FLT-PET study of Patient 2; Patlak-*P*'s contrast is higher than Patlak or SUV and appears slightly noisier. This heightened contrast results in better distinction of the tumor's caudal-most extent from the adjacent soft tissue. In addition, the enhanced contrast provided by Patlak-*P* improves the visualization of a small REU that is clearly present in both the Patlak and Patlak-*P* images but only subtly so in the SUV image. 

The examination of both slices shows that tumor boundaries are made sharper but also appear slightly noisier (due to background reduction) by applying Patlak-*P*, as evidenced by TB ratios: 2.04 (Patlak-*P*), 1.43 (Patlak), and 1.36 (SUV) for the tumor nodule and 4.639 (Patlak-*P*), 2.912 (Patlak), and 2.77 (SUV) for the tumor cross-section (second row of Figure 5). This observation also holds for anatomic structures in the vicinity of the tumor cross-section. However, this effect was less obvious for the prominent bony structures in the second row of Figure 5 (arrows), where Patlak-*P* provided better resolution without a similar increase in apparent noise. 

Visual analysis of Figure 6 indicates that Patlak and Patlak-*P* possess higher contrast and image quality than SUV for the tumor and the cerebellum. These visual findings do not reflect calculated TB ratios, which do not vary significantly between techniques: 1.71 (Patlak-*P*), 1.59 (Patlak), and 1.69 (SUV). TB_SUV_, however, is artificially inflated because, as before, the background region in the SUV image (arrow in Figure 6) is not well resolved. Similarly, SUV depicts the tumor with blurrier boundaries and does not delineate the teeth structure as well. Furthermore, cerebellar details (arrow in Figure 6) seen in Patlak and Patlak-*P* images are missing and/or blurred in the SUV image, adding further evidence that Patlak-*P* could have an impact in neurometabolism studies.

### 3.6. Overall Comparison

In the previous subsections, we performed qualitative and quantitative analyses of the most interesting features in each patient's FDG and FLT images. However, it is also important to provide an overall picture of how well each technique performs in terms of contrast and image quality. 

Table 1 was compiled from TB ratios calculated from the REUs using each technique. Paired, two-tailed Student *t*-tests showed that (a) TB ratios calculated by Patlak-*P* and Patlak were statistically similar (*P* = 0.125); (b) TB ratios calculated by Patlak and SUV were not statistically similar (*P* = 0.004); (c) TB ratios calculated by Patlak-*P* and SUV were not statistically similar (*P* = 0.002). 

 In general, visual contrast observed in Patlak-*P* and Patlak images were higher than SUV images. There was one instance where the Patlak image possessed significantly higher contrast than the Patlak-*P* image (2-FDG), and two instances where the Patlak-*P* image possessed significantly higher contrast than the Patlak image (2-FLT and 3-FLT). In all of these cases, images with significantly enhanced contrast appeared slightly noisier. 

Across all studies, SUV images possessed blurrier boundaries than their Patlak and Patlak-*P* counterparts, which impacted *delineation* of teeth, bony structures, the cerebellum, and REU/tumor boundaries. This effect was often emphasized around small structures (Figures 3(a)–3(c) and Figure 5), which appeared to be more resolved in Patlak and Patlak-*P* images.

## 4. Discussion

We performed this feasibility study to assess the possible impact of Patlak-*P*, a novel method for generating Patlak *K*_*i*_ parametric maps, on radiotherapy planning. Quantitative and qualitative comparisons demonstrated that Patlak-*P* images are similar to Patlak images and possess higher contrast, sharper boundaries, and (generally) better image quality than SUV images. These findings show that Patlak or Patlak-*P* images may have significant bearing on radiotherapy planning. In particular, improved resolution of tumors from surrounding structures may help dose-planning physicians deliver a lethal dose to tumors while preserving more normal tissue. 

### 4.1. The Challenge of Nonlinear Regression

Nonlinear regression is a more difficult problem than linear regression, because solutions of nonlinear regression are not guaranteed to be unique. This added variability increases the sensitivity of *λ* and was likely the proximate cause of discrepancies between the TB ratios of Patlak and Patlak-*P* in three studies. Since we only applied the Levenberg-Marquardt algorithm with a single initial guess in this study, determination of *λ* can be made more robust using several methods. The easiest method is to execute the Levenberg-Marquardt algorithm with multiple initial guesses and accept the parameter set with the lowest “energy” as the global minimum. In fact, it has been shown that dynamic PET studies benefit from this technique [29]. We do not anticipate needing more sophisticated methods such as simulated annealing [30] or genetic algorithms [31], because (a) the number of parameters (3) is small, and (b) each parameter is physical in nature. Thus, we should be able to develop ranges of initial guesses for each parameter that are both small and meaningful so that a fine-grid exhaustive search is computationally manageable. If additional refinement is needed, we will explore methods used to denoise dynamic PET data such as HYPR processing [32]. We expect that improving the estimation of *λ* will increase the statistical similarity (*P*-value) between Patlak and Patlak-*P*.

### 4.2. Choosing the Acquisition Start Time (*t*_0_)

Since this was a retrospective analysis, we could not choose *t*_0_. However, by choosing *t*_0_ to be approximately 15–20 minutes after-injection, as was the case in this feasibility study, the Patlak assumption of minimal dephosphorylation may be better upheld than if *t*_0_ was delayed further. For example, if *t*_0_ was chosen to be 2 hours after injection, even a small k_4_, depending on k_2_ and k_3_ values, may decrease the extrapolated uptake at infinity and, thus, underestimate *P*_*∞*_ as a correlate for *K*_*i*_. Therefore, if the objective is to mimic *K*_*i*_, then *t*_0_ = 15–20 minutes after-injection may be the most suitable. However, as is shown in several delayed-time PET publications [33, 34], if higher contrast is the goal (e.g, radiotherapy), then delaying acquisition (*t*_0_) until 30–60 minutes after-injection may be the better option.

### 4.3. Radiotherapy Planning

To our knowledge, there has not been a study on how using Patlak *K*_*i*_ parametric maps, rather than static images, would alter radiotherapy planning. Though, Visser et al. [19] do give a basis for such investigations. In this work, they show that biological tumor volume (BTV), as defined by a threshold of 50%, is significantly smaller in Patlak images than SUV images. However, since it has been shown that tumor volumes derived using thresholding techniques are suboptimal [35], it would be unwise to accept these findings as confirmation that dose-planning would be improved by using Patlak images. Based upon the results of this work, the work of Visser et al., and other investigations into the use of Patlak *K*_*i*_ maps in radiotherapy planning and monitoring [20, 21, 36], we believe such studies are warranted. Furthermore, Patlak-*P* would be better suited for this application than Patlak, because it is easier to implement [22] and less subject to variability; two advantages that would help facilitate multicenter clinical trials and routine implementation in the clinic.

### 4.4. Therapy Monitoring

In this preliminary work, our primary focus was an initial validation of Patlak-*P* for radiotherapy planning. However, Patlak-*P* has the potential to impact other areas of research and clinical projects. The first application area we propose, therapy monitoring, is closely associated with radiotherapy planning. Early indication of treatment efficacy can be very beneficial [2, 12–18]; if a treatment is not having the desired effect, then it may be substituted for another. 

Though SUV is the current standard in treatment monitoring, it has two major weaknesses: (a) it does not account for unmetabolized tracer, and (b) it crudely approximates the integrated input function with several normalization factors (e.g., the ratio of injected activity to body mass). The quantitative effect of these weaknesses on prediction of therapeutic outcome from serial measurements of SUV was assessed by Freedman et al. [14] using several posteriori corrections. First, they corrected for the unmetabolized tracer in SUV using the results of two-compartmental kinetic modeling. Second, they replaced the typical normalization factor with the integrated activity. Third, they corrected for both effects. In order, these corrections increased the correlation (*r*) between percent change in SUV (%SUV) and therapeutic outcome from 0.77 to 0.88, 0.85, and 0.97. Since the first correction should be equivalent to using SUV_*∞*_ instead of SUV, it would be logical to conclude that Patlak-*P* may have bearing on treatment monitoring. Furthermore, two investigations by Dimitrakopoulou-Strauss et al. [13, 18] and one study by Thie et al. [36] have shown that (a) outcome prediction may benefit from serial observations of Patlak *K*_*i*_ and SUV; (b) tumor staging may benefit from serial observations of Patlak *K*_*i*_ from two different tracers (^18^FDG and ^68^Ga-BZH3); (c) diagnosis of suspicious nodules may be improved by combining TAC slope and static uptake. Thus, it is possible that the combination of SUV and SUV_*∞*_ may improve the accuracy of outcome prediction and tumor staging relative to SUV. 

We emphasize here that it is the proportional relationship between *K*_*i*_ and *P*_*∞*_ that allows all observations, independent of tracer or application, made previously regarding SUV and *K*_*i*_ to remain partially or wholly true for SUV_*∞*_ and *K*_*i*_. For the unique case of FDG, which has well-characterized population-based input functions and minimal metabolites, *K*_*i*_ values may be directly calculated from *P*_*∞*_ and *λ* (see Appendix).

### 4.5. Neurometabolism Studies

Studies of neurometabolism, like radiotherapy planning, benefit from sharp boundaries, high contrast, and resolution of structural detail. These reasons alone suggest that Patlak-*P* may have a significant impact on neurometabolism studies. However, in contrast to radiotherapy planning, where the main goal is to provide high-quality images to the dose-planning physician, neurometabolism studies may require quantitative measures of uptake. Fortunately, these measures are typically not required to be absolute, only relative, because uptake patterns in certain regions of the brain (e.g., cerebellum) are often constant across wide populations. For example, Mosconi et al. [37] investigated how to best differentiate patients with Alzheimer's disease (AD) from normal patients using dynamic PET. They found that the two groups were best separated using cerebral metabolic rate (CMR_glc_) ratios between the medial temporal lobe (MTL) and pons and the posterior cingulate cortex (PCC) and pons. These relative metrics could easily be calculated using Patlak-*P*. Furthermore, the hallmark of AD is an atypical pattern of FDG uptake [38], not a global decline. Thus, Patlak-*P* may be able to improve the detection of early AD, because of its high contrast and ability to resolve structural details in the brain (Figures 3 and 6).

### 4.6. Extension to Multiple FOVs

In their work [10], Sundaram et al. demonstrated that simplified kinetic analysis methods may be extended beyond one FOV by using successive scans of several bed positions. In our view, the main obstacle to a similar extension for Patlak-*P* would be the determination of *λ*, because fewer time points would be available for analysis. However, this complication should have a minimal effect on accuracy, because the available time points will still span the same total amount of time (45 minutes). In this way, Patlak-*P* could be made more practical for targeted analyses of relatively large structures such as the brain or liver.

### 4.7. Future Studies

We believe that this feasibility study of a single cancer is sufficient to provide proof of principle of the Patlak-*P* technique and, thus, serve as the basis for planning future validation studies and clinical investigations. Validation studies are needed to compare (a) quantitative and qualitative similarities between *P*_*∞*_ and *K*_*i*_ parametric maps for other cancer types, (b) ratios of SUV_*∞*_, SUV, *K*_*i*_ (as calculated using Patlak-*P*, please see the Appendix), and *K*_*i*_ determined from FDG-PET measurements taken before and after treatment and (c) intrapatient ratios of *P*_*∞*_ and *K*_*i*_ between brain regions for the assessment of neurometabolism distributions.

## 5. Conclusions

In this feasibility study, we demonstrated a novel method (Patlak-*P*), which does not require any sampling data or an image-derived input function from the blood pool region, to generate parametric maps that mimic Patlak *K*_*i*_. Using quantitative and qualitative comparisons, we demonstrated that Patlak-*P* images are similar to Patlak images and possess higher contrast, sharper boundaries, and (generally) better image quality than SUV images. We believe that these improvements over SUV could have direct impact in radiotherapy planning, therapy monitoring, and neurometabolism studies.

## Figures and Tables

**Table 1:** TB ratios for all REUs analyzed. Headings under the study column reflect the patient's number and tracer type.

Study	Patlak-*P*	Patlak	SUV
1-FLT	3.448	3.773	2.977
	3.625	3.638	3.149
	2.537	2.617	2.163
	5.606	5.663	3.525
	1.280	1.541	1.127
	2.039	2.197	1.814
1-FDG	2.736	2.481	2.270
	1.543	1.506	1.411
2-FLT	5.329	3.932	3.451
	5.637	4.370	3.710
	1.555	1.417	1.289
2-FDG	1.922	2.661	1.952
	1.600	2.116	1.591
3-FLT	2.040	1.433	1.364
	4.639	2.912	2.774
	6.272	3.313	3.735
3-FDG	1.712	1.591	1.688
	2.237	2.234	2.116
	1.562	1.589	1.417

## Appendix

Here, we will present a detailed derivation of the Patlak-*P* equation, which was more briefly derived in Methods Section and outline how *K*_*i*_ may be calculated from *P*_*∞*_ for FDG. Again, we start with the Patlak equation

(A.1) $$\frac{P\left(t\right)}{\text{AIF}\left(t\right)}=K_{i}\frac{\int_{0}^{t}\text{AIF}\left(t^{\prime }\right)dt^{\prime }}{\text{AIF}\left(t\right)}+V.$$

Multiplying both sides by AIF(*t*) we obtain

(A.2) $$P\left(t\right)=K_{i}\overset{t}{\underset{0}{\int }}\text{AIF}\left(t^{\prime }\right)dt^{\prime }+V*\text{AIF}\left(t\right).$$

Now, we frame (A.2) to reflect the difference between activity (*P*) at initial time *t*_0_ and a later time *t*

(A.3) $$\begin{array}{c} P\left(t\right)-P_{0}=\left(K_{i}\overset{t}{\underset{t_{0}}{\int }}\text{AIF}\left(t^{\prime }\right)dt\prime +V\left(\text{AIF}\left(t\right)-\text{AIF}\left(t_{0}\right)\right)\right) \end{array}.$$

By applying the well-mixed assumption of the input function at late times, we obtain

(A.4) $$\begin{array}{cc} & P\left(t\right)-P_{0}\\ & =\left(K_{i}\overset{t}{\underset{t_{0}}{\int }}\text{AIF}\left(t_{0}\right)e^{-\lambda (t-t_{0})}dt\prime +V\left(\text{AIF}\left(t_{0}\right)e^{-\lambda (t-t_{0})}-\text{AIF}\left(t_{0}\right)\right)\right). \end{array}$$

By evaluating the integral and performing algebraic combination, we obtain

(A.5) $$P\left(t\right)=P\left(t_{0}\right)+\text{AIF}\left(t_{0}\right)\left(\frac{K_{i}}{\lambda }-V\right)\left(1-e^{-\lambda (t-t_{0})}\right).$$

It is not possible to extract the individual components of the uptake term,  AIF(*t*_0_)((*K*_*i*_/*λ*) − *V*), using nonlinear or linear curve fitting so we combine them into *α*:

(A.6) $$P\left(t\right)=P\left(t_{0}\right)+\alpha \left(1-e^{-\lambda (t-t_{0})}\right).$$

As shown previously in the Methods Section, *P*_*∞*_ is the sum of *P*(*t*_0_) and *α* and is directly proportional to *K*_*i*_ through the following equation:

(A.7) $$P_{\infty }=K_{i}\overset{\infty }{\underset{0}{\int }}\text{AIF}\left(t^{\prime }\right)dt\prime .$$

Thus, *K*_*i*_ may be calculated from *P*_*∞*_ if the total integrated arterial input function (*i*AIF_*∞*_) is accurately estimated. For FDG-PET, we propose two possible methods to improve patient-specific estimation of *i*AIF_*∞*_ relative to stand-alone, population-based input functions and, thus, increase the similarity between Patlak-*P* and Patlak (*K*_*i*_) ratios for serial studies.

The first option is implementing the population-based, triple-exponential input function developed by Vriens et al. [24]. This method showed good accuracy (*R* ^2^ = 0.856 for AUC) for a large patient population (*n* = 80) and does not require blood sampling or a dynamic cardiac acquisition. Furthermore, it may be possible to improve the accuracy of Vriens' model by using the Patlak-*Pλ* as the final exponential decay constant in the triple exponential.

The second option does not require blood samples but does require an initial dynamic scan over the heart from which an initial AIF will be derived. By using low-dose CT, the overall dose of this combined protocol should not be significantly changed relative to a single dynamic PET/CT acquisition. Similar to the first option, a triple exponential would be fit to the initial AIF, and the Patlak-*Pλ* would be used as the final decay constant.
